# Mutual reinforcement of pathophysiological host‐microbe interactions in intestinal stasis models

**DOI:** 10.14814/phy2.13182

**Published:** 2017-03-21

**Authors:** Ketrija Touw, Daina L. Ringus, Nathaniel Hubert, Yunwei Wang, Vanessa A. Leone, Anuradha Nadimpalli, Betty R. Theriault, Yong E. Huang, Johnathan D. Tune, Paul B. Herring, Gianrico Farrugia, Purna C. Kashyap, Dionysios A. Antonopoulos, Eugene B. Chang

**Affiliations:** ^1^ Department of MedicineUniversity of ChicagoChicagoIllinois; ^2^ Department of SurgeryUniversity of ChicagoChicagoIllinois; ^3^ Department of Cellular and Integrative PhysiologyIndiana University School of MedicineIndianapolisIndiana; ^4^ Enteric NeuroScience ProgramDivision of Gastroenterology and HepatologyMayo Clinic RochesterRochesterMinnesota; ^5^ Institute for Genomics and Systems BiologyArgonne National LaboratoryArgonneIllinois

**Keywords:** Gastrointestinal motility, gut microbiome, host‐microbe interactions, irritable bowel syndrome

## Abstract

Chronic diseases arise when there is mutual reinforcement of pathophysiological processes that cause an aberrant steady state. Such a sequence of events may underlie chronic constipation, which has been associated with dysbiosis of the gut. In this study we hypothesized that assemblage of microbial communities, directed by slow gastrointestinal transit, affects host function in a way that reinforces constipation and further maintains selection on microbial communities. In our study, we used two models – an opioid‐induced constipation model in mice, and a humanized mouse model where germ‐free mice were colonized with stool from a patient with constipation‐predominant irritable bowel syndrome (IBS‐C) in humans. We examined the impact of pharmacologically (loperamide)‐induced constipation (PIC) and IBS‐C on the structural and functional profile of the gut microbiota. Germ‐free (GF) mice were colonized with microbiota from PIC donor mice and IBS‐C patients to determine how the microbiota affects the host. PIC and IBS‐C promoted changes in the gut microbiota, characterized by increased relative abundance of *Bacteroides ovatus* and *Parabacteroides distasonis* in both models. PIC mice exhibited decreased luminal concentrations of butyrate in the cecum and altered metabolic profiles of the gut microbiota. Colonization of GF mice with PIC‐associated mice cecal or human IBS‐C fecal microbiota significantly increased GI transit time when compared to control microbiota recipients. IBS‐C‐associated gut microbiota also impacted colonic contractile properties. Our findings support the concept that constipation is characterized by disease‐associated steady states caused by reinforcement of pathophysiological factors in host‐microbe interactions.

## Introduction

Chronic diseases are often established through self‐reinforcing pathophysiological events that are difficult to interrupt. For example, nearly 25% of the US population is affected by disorders of gastrointestinal (GI) motility. Constipation remains one of the most common symptoms caused by GI dysmotility, where 12–19% of the US population is affected (Higgins and Johanson 2004). Constipation is often chronic in nature, and the causes leading to this condition remains unclear. Recent studies suggest a role for gut microbiota in constipation associated with IBS (Chey et al. 2015). A few studies have identified specific microbial species that have been associated with IBS‐C (Malinen et al. 2005; Maukonen et al. 2006; Lyra et al. 2009; Parkes et al. 2012). Another study identified microbiota associated with a distinct subset of IBS patients that did not correspond to IBS subtypes defined by the Rome II criteria (Jeffery et al. 2012).

Dysbiosis in IBS‐C has also been associated with alterations in microbial metabolite production. For example, methane excretion has been reported to be significantly higher from the gut microbiota of patients with constipation‐predominant IBS compared to nonconstipated subjects while other studies have been unable to confirm these associations (Higgins and Johanson 2004; Suply et al. 2012; Vega et al. 2015). Other studies have shown that fecal microbiota from constipated patients produce increased sulfides and hydrogen, but less butyrate compared to healthy subjects (Chassard et al. 2012). Soluble fiber consumption is associated with a significant improvement of constipation, an effect attributed to increased fermentation and production of short‐chain fatty acids (SCFA) (Quigley 2011).

There is emerging evidence suggesting that disruption of the bidirectional interplay between GI motility and the gut microbiota contributes to the development of GI diseases (Barbara et al. 2005). For example, dietary intake impacts GI transit time, but can also affect gut microbial community structure and function. Slow transit time and constipation induced by pharmacological agents, such as loperamide, can lead to alterations in gut microbial communities (Kashyap et al. 2013). On the other hand, the direct effect of specific bacterial strains has also been shown to accelerate or reduce the activity of the host migrating myoelectric complex (MMC) (Husebye et al. 2001).

In these studies, we propose that the gut microbiota contributes to the development of constipation, which feeds forward to promote physiological changes and reinforces conditions that maintain gut dysbiosis – creating a chronic state that is difficult to interrupt. In our study, two models of constipation were used where germ‐free mice were colonized with either stool from specific pathogen‐free (SPF) mice with pharmacologically‐induced constipation (PIC) or from fecal microbiota from a patient with IBS‐C. Because the type of diet can cause initial variability in GI transit time, both of the groups transplantated with either PIC or IBS‐C microbiota were fed identical diets.

## Materials and Methods

### Animals

C57Bl/6 female and male SPF mice were bred and housed in the animal care facility at the University of Chicago. Mice of 8–10 weeks of age were treated with 0.1% loperamide in their drinking water for 7 days. For microbiota transfer experiments, 8‐ to 10–week‐old germ‐free mice were gavaged with 1.5 mL cecal homogenate from loperamide‐treated and nontreated donors, and fecal homogenate from IBS‐C patients and control samples. Intestinal colonization lasted for 3–4 weeks until mice were sacrificed. All protocols were approved by institutional IACUC.

### Human subjects

All human studies were approved by the institutional review board. A stool sample was collected from a patient with IBS‐C with delayed colonic transit as well as an age‐ and sex‐matched healthy control subject with normal transit time and stored frozen in −80°C freezer until the study. The patient with IBS‐C met Rome III criteria for constipation predominant IBS. The patient completed a 7‐day bowel diary and reported two to three bowel movements per week, which were all identified as Type 1 at the Bristol stool scale.

### Colonization experiments

Eight to 10‐week‐old germ‐free mice were gavaged with 1.5 *μ*L cecal homogenate from loperamide‐treated and nontreated donors, and fecal homogenate from IBS‐C patient and control subjects. Intestinal colonization lasted for 3–4 weeks until mice were sacrificed.

### Treatment and GI transit time measurements

Total GI transit time was determined as previously described (Nagakura et al. 1996). Briefly, mice were gavaged with a solution of 0.5% activated charcoal suspended in 1% methylcellulose (Sigma‐Aldrich). Total transit time was determined as the time interval between gavage of the dye and the first appearance of charcoal in fecal pellets.

### Bacterial DNA extraction

Mouse cecal samples were collected and homogenized in 1 mL extraction buffer (50 mmol/L Tris [pH 7.4], 100 mmol/L EDTA [pH 8.0], 400 mmol/L NaCl, 0.5% sodium dodecyl sulfate [SDS]), and 20 *μ*L proteinase K (20 mg/mL) containing 500 *μ*L of 0.1‐mm‐diameter zirconia/silica beads (BioSpec Products, Bartlesville, OK). Samples were placed in a Mini‐Beadbeater‐8 cell disrupter (BioSpec Products) for 5 min to lyse bacterial cells and incubated overnight at 55°C. Bacterial DNA extraction was performed with phenol/chloroform/isoamyl alcohol, and precipitation with ethanol. Isolated DNA was dissolved in nuclease‐free water.

### 16S rRNA‐based Illumina assay and data analysis

To assess bacterial community structure, primers specific for 16S rRNA V4‐V5 region (Forward: 338F: 5f‐GTGCCAGCMGCCGCGGTAA‐3G and Reverse: 806R: 5a‐ GGACTACHVGGGTWTCTAAT‐3 ) that contained Illumina 3 adapter sequences as well as a 12‐bp barcode were used. Sequences were generated by an Illumina MiSeq DNA platform at Argonne National Laboratory and analyzed with QIIME software (Caporaso et al. 2010). OTUs were picked at 97% sequence identity using cdhit and a representative sequence was then chosen for each OTU by selecting the most abundant sequence in that OTU. Sequences were aligned using PyNAST and taxonomy was assigned using the RDP Classifier. The PyNAST‐aligned sequences were also used to build a phylogenetic tree with FastTree and unweighted UniFrac distances then computed between all samples for additional ecological analyses.

### In vitro contractility measurements

Dissected proximal colons, totaling four per mouse, were cut into 0.5‐cm‐long circular rings. Colonic rings were attached to isometric force transducers and equilibrated for 1 h in a Krebs buffer‐containing organ bath (Kent Scientific). Rings were set at an optimal resting tension of 1.5 g, and then contracted with carbachol at concentrations of 5 × 10^−7^, 5 × 10^−6^, and 5 × 10^−5^ mol/L, or KCl at concentrations of 40 and 60 mmol/L. After each contraction, rings were washed and left to rest for 15–20 min before the next contraction was initiated.

### SCFA analysis

Short‐chain fatty acids (SCFAs), including acetate, propionate, and butyrate, were analyzed using Varian Saturn 2000 GC‐MS‐MS. SCFAs were extracted in diethyl ether but with slight modifications (Renom et al. 2001). Briefly, fresh fecal and cecal contents were collected and weighed in 1.5 mL Eppendorf tubes. Samples were thoroughly homogenized in 600 *μ*L of nuclease‐free water using a sterile pipette tip and centrifuged at 13,000 *g* for 10 min at room temperature. The supernatant (500 *μ*L) was transferred to a fresh Eppendorf and acidified with 100 *μ*L of 50% H_2_SO_4_. After acidification, 5 *μ*L of 3 mmol/L isobutyric acid was added as an internal standard. SCFAs were extracted by adding 500 *μ*L of diethyl ether, vortexing for 30 sec, and quickly centrifuging at 5000 *g*. The upper ether layer (500 *μ*L) was transferred to a fresh Eppendorf tube and the extraction was repeated twice. 1 mL of the extract was transferred to a glass vial and derivatized with 250 *μ*L of MTBS‐TFA. Samples were run on GC‐MS‐MS within 24 h.

### Biolog functional assays

Biolog GenIII is a set of colorimetric assays arrayed across a 96‐well plate, including 72 carbon substrates, such as sugars, carboxylic acids or amino acids, and 24 environmental sensitivity assays, such as various pH, salinity, and antibiotic treatments. Wells containing communities that perform better on particular assays undergo greater color change. Patterns of color change can be used to compare collective metabolic potential or stress tolerance across experimental communities, providing a robust measure of microbiome function as a multivariate emergent property. Equal amounts of cecal contents were homogenized in a microcentrifuge tube containing 1 mL of Biolog Inoculating Fluid‐A (IF‐A). Particulates were pelleted by spinning tubes for ~5 sec in a mini‐centrifuge, and 100 *μ*L supernatant were transferred to a tube containing 10 mL IF‐A. The inoculum was vortexed, transferred to a multi‐channel pipette reservoir, and added at 100 *μ*L per well. Plates were incubated for 24 h, and absorbance at 590 nm was evaluated photometrically to measure tetrazolium violet dye reduction, a proxy for per well microbial metabolic activity. Using the multivariate statistical package PC‐ORD to analyze absorbance values, two‐way cluster dendrograms were generated illustrating the relationship between samples. Indicator analysis was performed to identify which substrates significantly differed between treatments, driving the divergent relationship in functional signatures. Principle Coordinate Analysis (PCoA) of Biolog data was performed in QIIME using the Bray–Curtis similarity index.

### Statistical analysis

Microbial community *β*‐diversity was analyzed by Anosim test and ANOVA test. Biolog data was analyzed by the MRPP test. The remaining data were analyzed by Student's *t*‐test with *P* < 0.05 was considered statistically significant.

## Results

### SPF mice have significantly shorter GI transit time when compared to GF mice, and show lower sensitivity to carbachol and KCL

**Figure 1:**
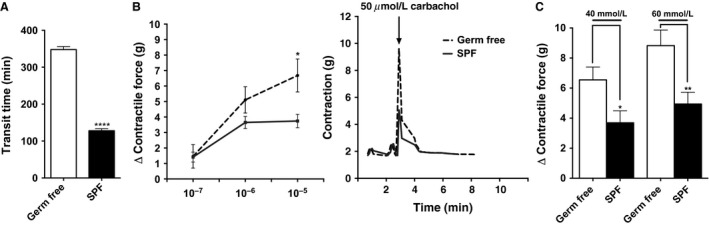
GF mice have increased GI transit time and altered contractility when compared to SPF mice. (A) Whole gut transit time of the GF and SPF mice. (B) *Left:* quantification of changes (Δ) in peak force produced by colonic rings from SPF and GF mice in response to different amounts of carbachol. *Right:* representative tension recordings from colon rings contracted by 50 *μ*mol/L carbachol stimulation. (C) quantification of changes (Δ) in peak force produced by colonic rings from GF and SPF mice in response to KCl. In (A) *n *=* *3 per group presented as mean ± SEM, **** *P *<* *0.0001. In (B) and (C) data shown represents the relative force of the rings from proximal parts of the colon. Each bar represents the mean ± SEM 4 different mice 4 rings, *n *=* *16 *P <* *0.05, **P <* *0.01. GF, Germ‐free; SPF, specific pathogen‐free.

To evaluate the effect of the gut microbiota on GI transit time, we compared transit time in mice lacking any gut microbiome – germ free (GF) mice, to those with a normal gut microbiome – SPF mice. Whole gut transit times (Fig. 1A) in GF mice were significantly longer than in SPF mice, as measured by the appearance of charcoal in stool following gavage (136 min vs. 340 min, *n *=* *3–5, ****P <* *0.0001), indicating delayed whole gut motility in the GF mice.

To better understand the role of gut microbes in maintaining normal GI motility, we measured the contractile responses of colonic rings from GF and SPF mice to various contractile agonists (carbachol and KCl) ex vivo. The proximal colon of each group was divided into four segments. The GF colonic rings showed higher sensitivity to 50 *μ*mol/L carbachol (6.4 g vs. 3.5 g, *n *=* *16, *P <* *0.05), and both 40 mmol/L (6.5 g vs. 3.7 g, *n *=* *16 per group, *P <* *0.05) and 60 mmol/L (8.8 g vs. 4.9 g, *n *=* *16 per group, **P <* *0.01) KCL compared to colonic rings from SPF mice (Fig. 1B and C). These findings are consistent with hyper‐reactive colonic phenotype after antibiotic treatment (Muller et al. 2014), and indicate that gut microbes can significantly impact the gut neuromuscular apparatus responses.

### Pharmacologically‐induced constipation (using loperamide) significantly alters gut microbial community structure

**Figure 2:**
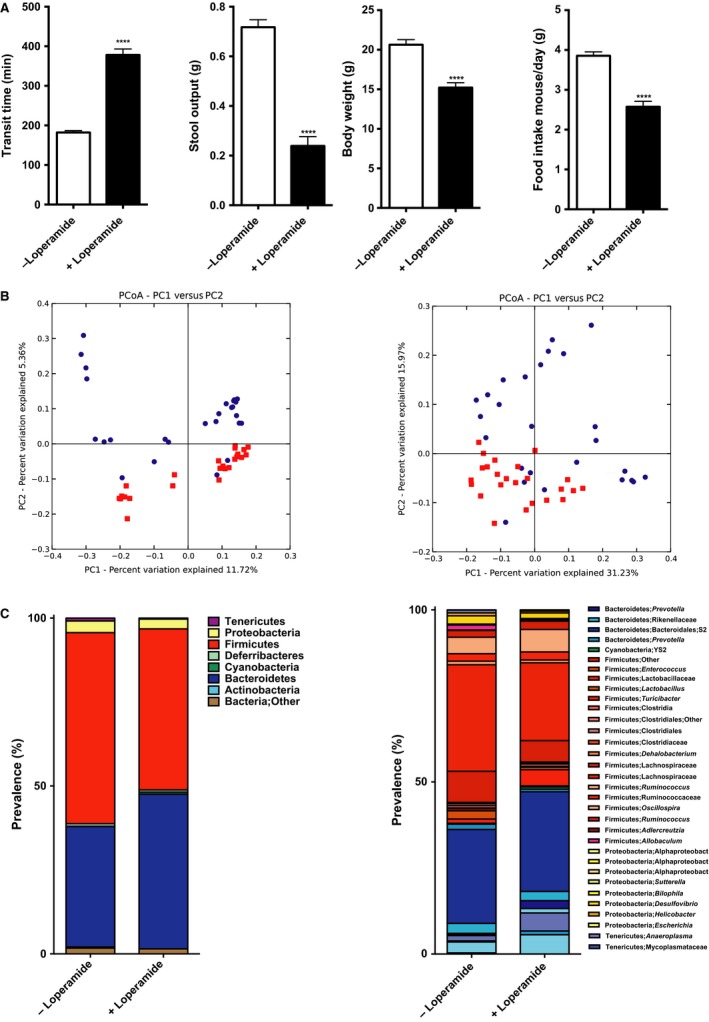
Loperamide treatment induces constipation leading to taxonomic changes in microbial community. (A) Whole gut transit time at the day of sacrifice (*n *=* *25–27 in each group), stool output over 16 hours during dark cycle (*n *=* *8 in each group), body weight at the day of sacrifice (*n *=* *27 in each group), and daily food intake over the course of treatment (*n *=* *18 in each group). (B) PCoA plot showing unweighted (left) and weighted (right) UniFrac from the mice cecal contents, *n *=* *24–27 in each group, *P *=* *0.01, *R* = 0.12 (unweighted), *P *=* *0.01, *R* = 0.22 (weighted). Red squares represent control group, and blue circles – loperamide‐treated mice. (C) *Left:* phyla distribution of cecal microbiota. *Right:* microbial class/family shifts between control and loperamide‐treated group. In (A) data presented as mean ± SEM, *P <* *0.05, **** *P *<* *0.0001.

To determine the effects of slow transit on the microbial composition and function, adult C57Bl/6J SPF mice were treated either with loperamide (0.1% in water) or maintained on regular water for 7 days. Whole gut transit times (Fig. 2A) in mice with pharmacologically induced constipation (PIC) were significantly longer than those in control mice, as measured by charcoal appearance in stool (182 min vs. 378 min, *n *=* *25–27, ***P <* *0.001). There was also a significant decrease in stool output observed after treatment with loperamide at the day of the sacrifice (0.7 g vs. 0.24 g, *n *=* *8 per group, ****P <* *0.0001) (Fig. 2A). Loperamide has been shown to decrease body weight due to decreased food intake in rodents. In our study, we observed decreased body weight (20.6 g vs. 15.2 g, *n *=* *27 per group, ****P <* *0.0001) and decreased food intake (3.6 g/mouse/day vs. 2.6 g/mouse/day) after the treatment (Fig. 2A, left panel).

There were significant differences in cecal microbial communities among mice treated with loperamide and control mice based on 16S rRNA marker gene sequencing using the MiSeq Illumina platform. Significant changes in *β*‐diversity using both unweighted and weighted UniFrac distance metrics were observed in mice with PIC, consistent with changes in microbial community richness and evenness, respectively, (*n *=* *24–27 per group, *P *<* *0.05, Anosim test) (Fig. 2B left and right panels). We did not observe significant differences in *α*‐diversity between two communities using Shannon diversity index (data not shown). Taxonomic analysis revealed significantly increased representation of the phyla Bacteroidetes and significantly decreased Firmicutes in the loperamide‐treated mice (Fig. 2C left panel), corroborating the findings from loperamide‐treated humanized mice (Kashyap et al. 2013). Further analysis beyond the phyla level in our samples showed most significant increase in families – Prevotellaceae, Porphyromonadaceae, and Bacteroidaceae within Bacteroidetes, and a decrease in Ruminococcaceae and Lachnospiraceae family members from Clostridia class within Firmicutes (Fig. 2C) in the loperamide‐treated mice. PIC mice showed increased representation by Bacteroidales family *S‐24‐7*,* Parabacteroides distasonis* and *Bacteroides ovatus* species, suggesting that stasis provides a beneficial environment for these species.

### Colonization of GF mice with cecal microbiota from PIC mice results in slower GI transit

**Figure 3:**
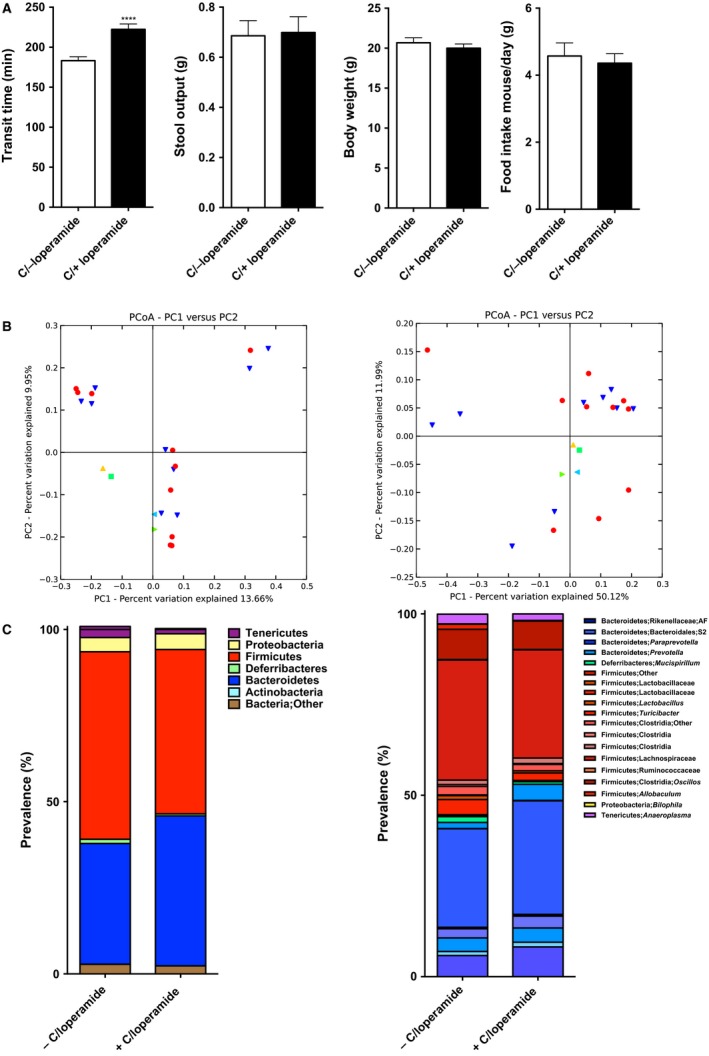
Colonization of Germ‐free mice with contents from loperamide‐treated mice increases GI transit time when compared to recipients of nontreated mice, but shows no significant taxonomic differences between two groups. (A) Whole gut transit time at the day of sacrifice (*n *=* *17–19 in each group), stool output over 16 hours during dark cycle (*n *=* *7–8 in each group), body weight at the day of sacrifice (*n *=* *16–17 in each group), and daily food intake over the course of treatment (*n *=* *7–8 in each group). (B) PCoA plot showing unweighted (left) and weighted (right) UniFrac from the colonized mice cecal contents, *n *=* *10 in each group, *P *=* *0.67, *R* = −0.05 (unweighted), *P *=* *0.01, *R* = 0.23 (weighted), Anosim test. Red circles represent mice conventionalized with contents from control group donors, yellow, and green triangle – control donors, green square, and blue triangle ‐ PIC donors. (C) *Left:* phyla distribution of cecal microbiota from colonized mice. *Right:* microbial family distribution. In (A) data presented as mean ± SEM, ****P <* *0.0001.

To determine whether PIC‐associated changes to the gut microbial community in turn affect host function, 12‐ to 14‐week‐old GF mice were colonized with cecal contents from SPF control or SPF PIC mice, and were not receiving loperamide after colonization. Four weeks after colonization, mice that received cecal contents from PIC mice showed significantly longer GI transit times compared to those receiving control cecal contents (222 min vs. 183 min, *n *=* *17–19 per group, ****P <* *0.0001) (Fig. 3A). Mice from two groups of recipients showed no differences in stool output (0.68 g vs. 0.69 g, *n *=* *7–8 per group), changes in body weight (20.7 g vs. 20 g, *n *= 16–18 per group), or food intake (4.6 g/mouse/day vs. 4.4 g/mouse/day, *n *=* *7–8 per group) (Fig 3). These data therefore demonstrate that the gut microbial communities changed upon the development of intestinal stasis, and that the functional phenotype could be transferred to recipient mice.

We did not observe significant differences in *α*‐diversity between the two groups. Anosim test of *β*‐diversity did not show significant differences unweighted (*P *=* *0.65) distance metrics, but showed significant differences in weighted (*P *=* *0.01) UniFrac distance metrics (Fig. 3B left and right, respectively), indicating differences in the abundance of bacterial community between two groups. The microbial profile of GF mice colonized with control microbiota was similar to that seen in SPF mice (Compare Figs. 2C to 3C). Similarly, the microbial profile of SPF mice treated with loperamide was similar to that of GF mice colonized with microbiota from loperamide‐treated mice (Compare Figs. 2C to 3C). These data indicate that microbial changes induced by chronic constipation can themselves slow GI transit time independent of any direct pharmacological effects of loperamide on GI transit.

**Figure 4:**
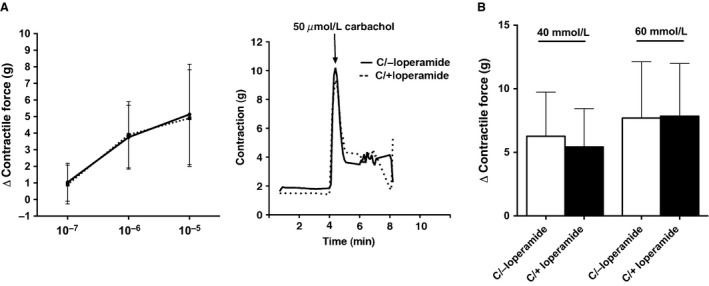
Colon rings from colonized mice show no significant differences between recipients of control and loperamide‐treated microbiota after carbachol and KCl stimulation. (A) *Left:* quantification of changes (Δ) in peak force produced by colonic rings from colonized mice in response to different amounts of carbachol. *Right:* representative tension recordings from colon rings contracted by 50 *μ*mol/L carbachol stimulation. (B) quantification of changes (Δ) in peak force produced by colonic rings from colonized mice in response to KCl. Data shown represents the relative force of the rings from proximal parts of the colon. Each bar represents the mean ± SEM 4 rings 6 different mice, *n *=* *24.

To determine whether the slower GI transit time in GF mice colonized with cecal contents from PIC donors was due to local alterations in colon contractility, we stimulated colonic rings from control and PIC colonized mice with carbachol and KCl. We did not observe differences in contractile properties between recipients of PIC microbiota and controls (*n *=* *24 per group; *P *=* *0.05; *t*‐test) (Fig. 4A, B). Taken together, PIC gut microbiota significantly decreased transit time while maintaining a similar microbial structure to the donors but did not affect contractility.

### Functional profiles of bacteria in PIC mice differ from control animals

**Figure 5:**
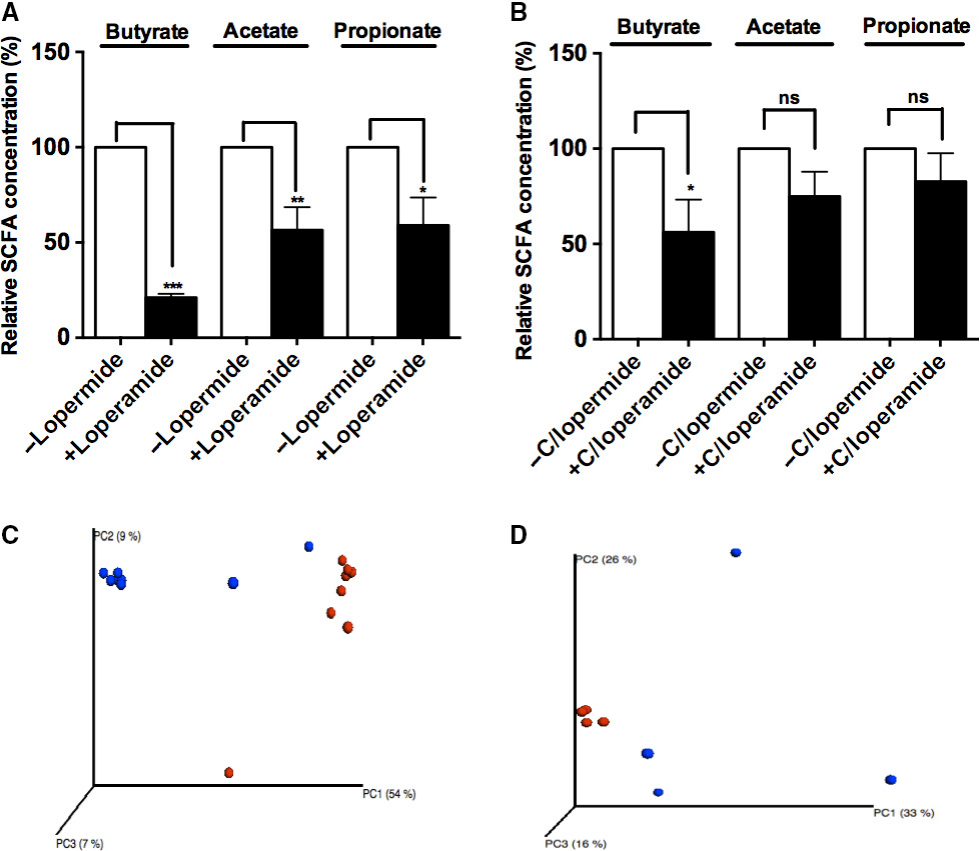
Loperamide treatment shifts microbial community metabolism that is persistent after microbiota transfer. (A) Butyrate, acetate and propionate relative concentrations in the cecal contents of control and loperamide‐treated mice. (B) SCFA concentrations of the cecal microbiota collected from colonized recipients with cecal microbiota from control and loperamide‐treated mice after 3–4 weeks of colonization. (C) PCoA analysis of community metabolism in PIC and control samples using Biolog. Data represents substrate metabolism in aerobic and anaerobic conditions, and separation by substrates (*n *=* *8, *P *<* *0.00067, MRPP test). (D) PCoA analysis of community metabolism in PIC and control samples (*n *=* *4, *P *<* *0.0083, MRPP test). Blue dots – control, red dots – PIC samples. Values are expressed as percentage of SCFA levels in cecal contents obtained from control mice, *n *=* *8 mice per group, *P <* *0.05, **P <* *0.01, ***P <* *0.001, ns, not significant; SCFA, short‐chain fatty acids.

Although the GI transit time was decreased in both mice colonized with control and with PIC cecal contents, the latter showed a significantly lower recovery of GI motility. These findings show that two communities are significantly different by DNA analysis, and suggest that the functional properties of the microbiota from the two groups are different as well. To examine this possibility, we performed a targeted screen of the functional and metabolic profiles of the microbiota from PIC and control mice. Butyrate has been shown to improve GI motility by promoting cholinergic and nitrenergic neuron development (Kamath et al. 1987; Chevalier et al. 2010), and is produced by the Clostridiales taxa, which was present in lower relative abundance in the cecal samples from PIC mice. Butyrate, propionate, and acetate levels were all significantly decreased in cecal samples (21%, 57% and 59%, respectively, *n *=* *8 per group, *P <* *0.05, **P <* *0.01, ***P <* *0.001) from PIC mice compared to controls (Fig. 5A). Decreased abundance of Clostridiales may lead to decreased butyrate production in constipated mice and this might be a feed‐forward mechanism leading to exacerbated constipation. However, studies show that propionate and acetate also can modulate colonic propulsion (Hurst et al. 2014), and it is possible that decreased propionate and acetate levels promote delayed intestinal transit. Additionally, SCFA levels in GF mice colonized with microbiota from PIC mice showed significant decrease in butyrate levels (56%, *n *=* *8, *P <* *0.05), and a trend toward decreased acetate and propionate levels. However, these latter changes were not statistically different (Fig. 5B).

We also used the Biolog GenIII system to directly measure the functional (metabolic) profiles of cecal microbiota from PIC and control mice, and recipient mice of PIC and control microbiota. Clear separation between PIC and control mice by anaerobic and aerobic substrates, as well as specific substrates in these groups, was observed by PCoA ordination plots. These findings indicate divergent functional capacity of the communities (*n *=* *8, *P *<* *0.00067, MRPP test), similar to that observed in 16S rRNA analysis (Figs. 5C, 2C). Indicator analysis revealed that the microbial communities isolated from loperamide‐treated cecal contents contain microbial communities that were more active in metabolizing amino acids, carboxylic acids, hexose acids, and different types of sugars when compared to control group. These functional differences may be due to a stasis‐induced shift in the microbial community.

PCoA ordinations from colonized PIC and control mice also exhibited significant separation between the two groups by anaerobic and aerobic substrates and indicators (*n *=* *4, *P *<* *0.0083, MRPP test) (Fig. 5D). Sugars were most actively metabolized by mice recipients colonized by donor PIC microbiota (Fig. 3B). These data show that the microbiota from PIC recipients, in combination with differences in bacterial community abundance, have distinct functional properties that affect on transit time.

### Microbiota transfer of fecal microbiota from a constipation‐predominant IBS patient into GF mice recapitulates slow GI transit and increased contractile responses to carbachol and KCL

**Figure 6:**
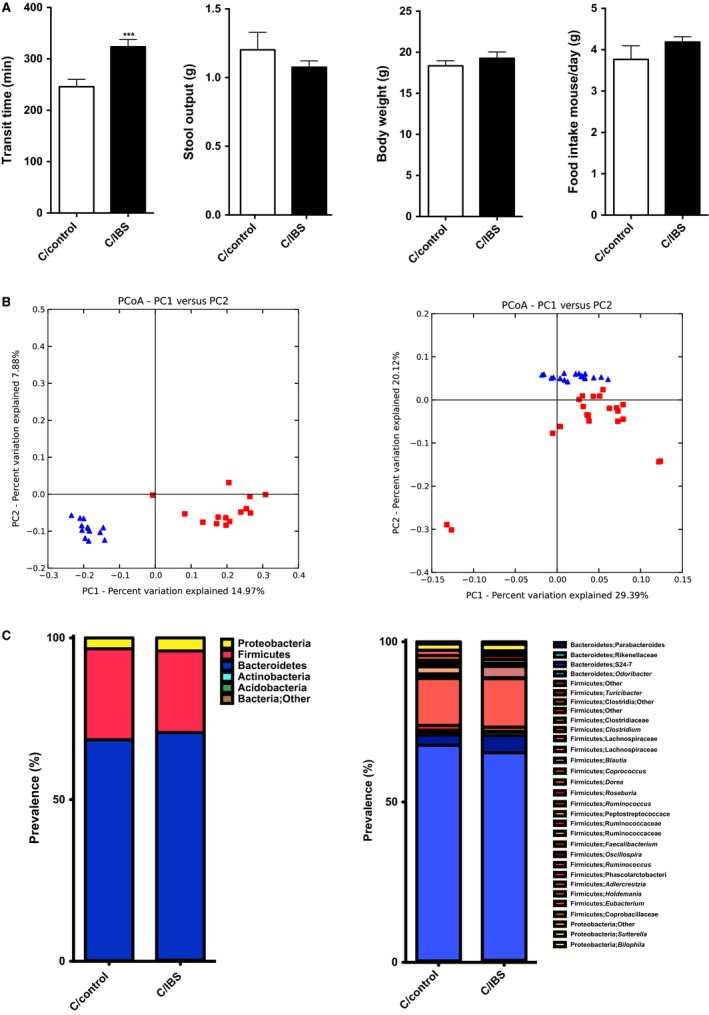
Colonization of Germ‐free mice with contents from constipation‐predominant IBS increases GI transit time when compared to control group, and shows significant taxonomic differences between two groups. (A) Whole gut transit time at the day of sacrifice, stool output over 16 hours during dark cycle, body weight at the day of sacrifice, and daily food intake over the course of treatment (*n *=* *11 in each group). (B) PCoA plot showing unweighted (left) and weighted (right) UniFrac from the cecal contents of mice colonized with IBS‐C and control samples. Red squares represent mice conventionalized with contents from control group donor, and blue triangles – mice conventionalized with IBS‐C donor. *n *=* *11 in each group, *P *=* *0.01, R = 0.75 (unweighted), *P *=* *0.01, R = 0.50 (weighted), Anosim test. (C) *Left:* phyla distribution of cecal microbiota from mice colonized with control and IBS‐C donor samples. *Right:* microbial family distribution. In (A) data presented as mean ± SEM, ***P <* *0.001.

To determine whether slow transit is recapitulated with microbiota from IBS‐C, we colonized GF mice with fecal contents from an IBS‐C patient and an age/gender‐matched control subject. 4 weeks after colonization, IBS‐C microbiota recipient mice showed significantly longer GI transit times compared to recipients of control microbiota (324 min vs. 246 min, *n *=* *11 per group, ***P <* *0.001) (Fig. 6A). There were no differences in stool output (1.2 g vs. 1.1 g, *n *=* *11 per group), body weight (18.4 g vs. 19.3 g, *n *=* *11 per group), and food intake (3.8 g/mouse/day vs. 4.2 g/mouse/day) between recipients of IBS‐C and control microbiota (Fig 6A). Similar to PIC microbiota recipients, IBS‐C microbiota recipients exhibited slower transit time but no decrease in stool output, body weight, or food intake compared to controls.

16S rRNA marker gene analysis of microbial communities of mice that had been colonized by IBS‐C and control microbiota showed no significant differences in *α*‐diversity, but significant differences in *β*‐diversity unweighted (*P *=* *0.01) and weighted (*P *=* *0.01) UniFrac distances (Fig. 6B left and right). Similar to PIC model, Bacteroidetes were more abundant in samples with slow transit (Fig. 6C). Statistical analysis showed that *Bacteroides uniformis*,* B. ovatus*,* P. distasonis* species were significantly higher in mice with slow transit. More importantly, *B. ovatus* and *P. distasonis* were also more abundant in PIC donors and recipients, suggesting that these two stains might have a role in promoting intestinal stasis.

**Figure 7:**
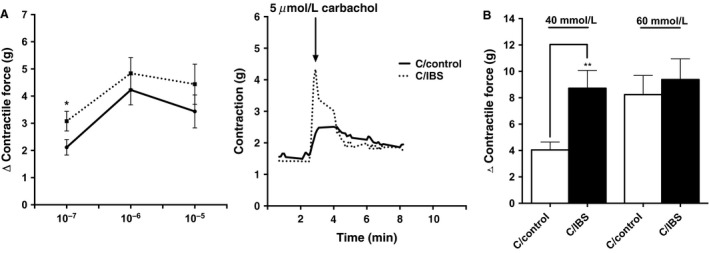
Colon rings from mice colonized with stool sample from IBS‐C show increased sensitivity to carbachol and KCl stimulation when compared to control group. (A) *Left:* quantification of changes (Δ) in peak force produced by colonic rings from colonized mice in response to different amounts of carbachol. *Right:* representative tension recordings from colon rings contracted by 5 μM carbachol stimulation. (B) quantification of changes (Δ) in peak force produced by colonic rings from colonized mice in response to KCl. Data shown represents the relative force of the rings from proximal parts of the colon. Each bar represents the mean ± SEM 4 rings 4 different mice, *n *=* *16, *P <* *0.05.

To determine whether slow transit in IBS‐C recipients is due to local alterations in colon contractility, we stimulated colonic rings from controls and IBS‐C recipients with carbachol and KCl. Similar to GF mice, recipients of IBS‐C with slow transit were more sensitive to carbachol (3.1 g vs. 2.1 g, *n *=* *16 per group; *P <* *0.05) and KCl (8.7 g vs. 4.0 g, *n *=* *16 per group; **P <* *0.01) (Fig. 7A, B). Our results show that IBS‐C gut microbiota significantly decreases transit and leads to colonic ring hypercontractility.

### Functional profiles of gut bacteria from GF mice humanized with IBS‐C and control subjects are significantly different

**Figure 8:**
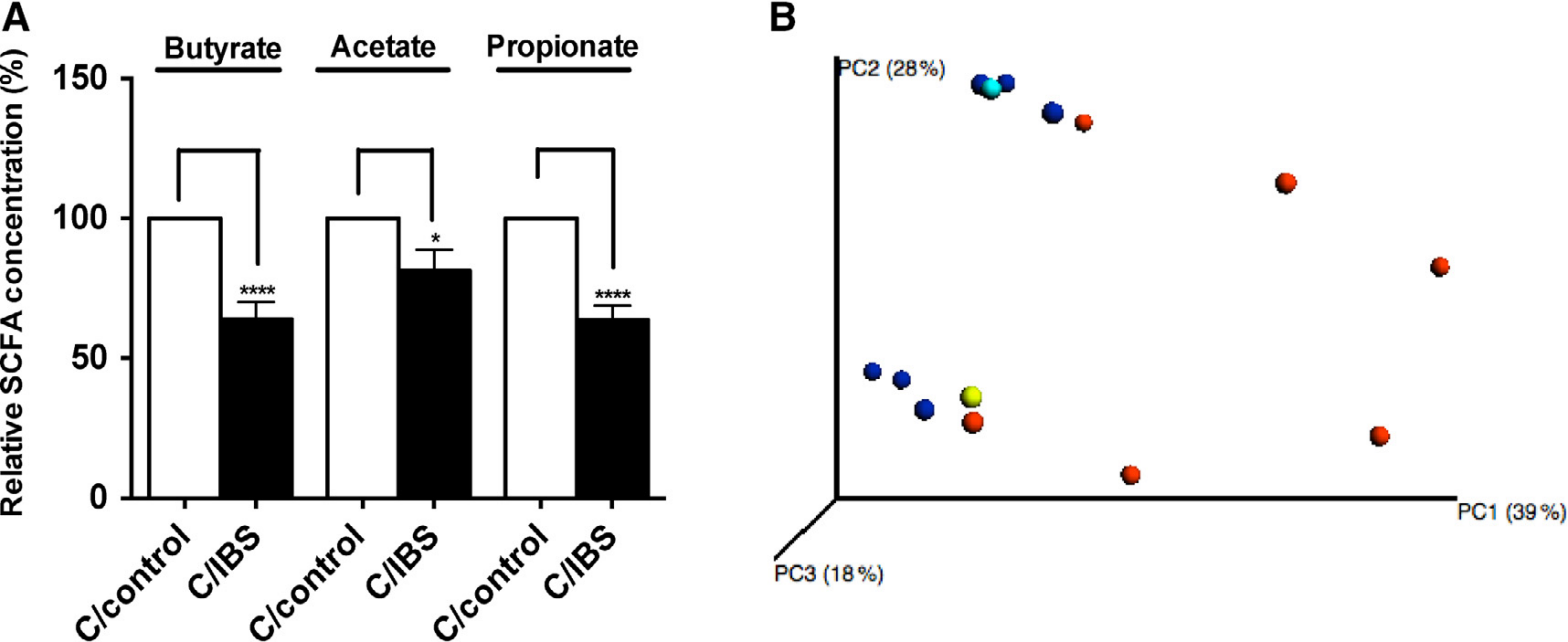
Microbial community metabolism in Germ‐free mice recipients of control and IBS‐C samples 4 weeks postcolonization shows significant differences between two groups. (A) SCFA relative concentrations in cecal microbiota from mice colonized with IBS‐C and control microbiota. (B) PCoA analysis of community metabolism using Biolog of IBS‐C and control recipients. Values are expressed as percentage of SCFA levels in cecal contents obtained from control mice, *n *=* *13 mice per group, *P <* *0.05, ****P <* *0.0001. SCFA, short‐chain fatty acids.

To examine if SCFA levels are different in IBS‐C and control microbiota recipients we screened cecal contents for SCFA. Mice displayed significant decreases of butyrate, propionate, and acetate levels (64%, 81% and 64%, *n *=* *11 per group, *P <* *0.05, ****P <* *0.0001) in IBS‐C recipients compared to controls (Fig. 8A, B).

We also used the Biolog GenIII assay to assess metabolic activity in control and IBS‐C GF recipient mice. Clear separation between recipients of IBS‐C and control microbiota (*n *=* *6, *P *<* *0.0075, MRPP test) could be seen with PCoA ordination plots (Fig. 8C). Indicator analysis revealed that the cecal microbial microbiota of IBS‐C recipients more actively metabolized amino acids, carboxylic acids, hexose acids, and sugars when compared to control recipients. The IBS‐C recipients exhibited a similar pattern to loperamide‐treated mice.

## Discussion

Our findings support the concept that chronic diseases such as constipation may be caused by steady states achieved through reciprocating reinforcement of pathophysiological host and microbial factors. This study shows that decreased gut motility alters the gut microbial community and its metabolism, leading to the promotion of the feed‐forward loop that slows GI transit time even further. The reinforcement of the decreased gut motility, in turn, sustains the selective advantage of constipation‐associated microbiota.

Disease‐promoting steady states have been observed in conditions such as obesity and inflammation. Microbiota from obese and lean twins induced obesity‐associated or lean metabolic phenotypes in GF mice (Ridaura et al. 2013). Gut microbiota also affect the progression of inflammation (Honda and Littman 2012). The unfavorable combination of inflammation and host genetics promotes development of gut dysbiosis and reduced diversity in the microbial community (Craven et al. 2012; Machiels et al. 2014). A bidirectional relationship between an altered microbial community and the host perpetuates an inflammatory state that leads to further effects on the immune response and maintains states of chronic inflammation.

Our findings also suggest that a bidirectional relationship between the gut microbiota and the host maintains chronic states of constipation. Several studies have shown that GI motility can dramatically affect gut microbial assemblage. Disrupted MMCs, which is associated with decreased housekeeping functions to sweep the small intestine between meals, is believed to contribute to the development of bacterial overgrowth (Pimentel et al. 2002). Conversely, intestinal microbiota have a significant role in the development and maintenance of intestinal motor functions. Bacterial metabolites directly stimulate gut enteric neurons, intestinal smooth muscle, and enteroendocrine cells that secrete biologically active peptides (Barbara et al. 2005). Colonization of GF mice with microbiota from loperamide‐treated or nontreated mice, as well as from IBS‐C or control patients improves GI transit time, indicating the importance of the microbiome on gut motility. However, GF mice receiving microbiota from loperamide‐treated mice exhibited slower GI transit time as compared to GF mice receiving microbiota from untreated SPF mice, suggesting that constipation leads to selection for certain types of gut microbes that impair GI motility. To test the effects of gut microbiota on GI motility, we also colonized GF mice with samples from IBS‐C and control subjects. 16S rRNA gene analysis showed that constipation in both models lead to increased abundance of *B. ovatus* and *P. distasonis* strains. In the PIC model, we observed significant decrease in Clostridiales class members while in IBS‐C, the decrease was less pronounced. These findings suggest that increases in these two strains potentially contribute to the development of constipation.

We observed decreased SCFA levels in recipients of PIC and IBS‐C microbiota. As the SCFA butyrate accelerates GI transit and plays an important role in neuronal development (Kamath et al. 1987; Chevalier et al. 2010; Rodolphe Soret et al. 2010), decreased levels of SCFAs could be a contributing factor for delayed transit time in PIC and IBS‐C GF recipients. The lack of a detectable difference in the contractility of isolated colonic rings isolated from mice that received microbiota from control or PIC would argue that the microbiota have not resulted in alterations in the intrinsic contractile machinery of colonic smooth muscle cells. This would however be consistent with microbial metabolites having a direct effect of regulating smooth muscle cell contractility, as this would not be evident in in vitro preparations. In contrast, colonic rings isolated from GF mice receiving microbiota from IBS‐C patients displayed increased sensitivity to contractile agonists while exhibiting a delayed transit time compared to mice receiving microbiota from control patients (Fig 7). Together, these data suggest that microbiota from IBS‐C patients can lead to stable alterations in colonic smooth muscle cells, perhaps resulting from higher levels of inflammatory cytokines in these patients.

Our findings also show that the colonization of GF mice with PIC‐cecal and IBS‐C fecal microbiota decreases GI transit time compared to mice receiving control cecal microbiota, suggesting that the microbiota, even 4 weeks after colonization, have residual effects on gut motility. These findings suggest that disruption of the self‐reinforcing cycle of host‐microbe interactions is important for gut motility, and can contribute to the development of chronic constipation. This knowledge can be leveraged to develop effective and sustained interventions for the prevention and treatment of disorders such as chronic constipation.

Based on our findings, effective therapy to treat chronic constipation could involve breaking the pathological cycle and establishing a new health‐promoting steady state. Selection against potential disease‐promoting gut microbiota could be achieved with dietary manipulation, specific antibiotics, prebiotics, and probiotics. Alternatively, bioactive metabolites produced by gut microbiota that promote gut motility could be administered as supplements. Consistent with these approaches, many constipation‐predominant IBS patients benefit from treatment with oral rifaximin therapy. Discontinuation of agents that promote stasis (medications, opiates, etc.) can also break the cycle and restore balance, a notion supported by our conventionalization studies where the constipating effects of PIC microbiota appear to diminish in the absence of loperamide. Finally, therapies directed toward enhancing motor and neuronal activity of the bowel can interrupt the cycle of feed‐forward factors causing chronic constipation. Prokinetic drugs that activate serotonin receptors, increase acetylcholine concentrations or inhibit acetylcholinesterase, an enzyme that metabolizes acetylcholine, enhance GI motility and benefit chronic constipation. Our study showed that constipated mice have lower levels of SCFA in cecal contents when compared to control mice, thus increasing bioavailability of SCFAs with probiotic treatment and increased fermentable, oligo‐, di‐, monosaccharides and polyols (FODMAP) diet could be an additional beneficial treatment for eliminating chronic constipation. Treatment with agents that alter fluid and electrolyte transport, such as chloride channel activators, may also affect the gut microbiota in a way that breaks the cycle of constipation‐promoting events between host and the microbe. The more we understand host‐microbe interactions that regulate GI motility, the better we will be able to identify treatments that alleviate GI distress.

## Conflict of Interest

None declared.

## References

[phy213182-bib-0001] Barbara, G. , V. Stanghellini , G. Brandi , C. Cremon , G. Di Nardo , R. De Giorgio , et al. 2005 Interactions between commensal bacteria and gut sensorimotor function in health and disease. Am. J. Gastroenterol. 100:2560–2568. 16279914 10.1111/j.1572-0241.2005.00230.x

[phy213182-bib-0002] Caporaso, J. G. , J. Kuczynski , J. Stombaugh , K. Bittinger , F. D. Bushman , E. K. Costello , et al. 2010 QIIME allows analysis of high‐throughput community sequencing data. Nat. Methods 7:335–336. 20383131 10.1038/nmeth.f.303 PMC3156573

[phy213182-bib-0003] Chassard, C. , M. Dapoigny , K. P. Scott , L. Crouzet , C. Del'homme , P. Marquet , et al. 2012 Functional dysbiosis within the gut microbiota of patients with constipated‐irritable bowel syndrome. Aliment. Pharmacol. Ther. 35:828–838. 22315951 10.1111/j.1365-2036.2012.05007.x

[phy213182-bib-0004] Chevalier, J. , P. De Coppet , G. Poupeau , P. Derkinderen , J. P. Segain , and M. Neunlist . 2010 Short‐chain fatty acids regulate the enteric neurons and control gastrointestinal motility in rats. Gastroenterology 138:1772–1782. 20152836 10.1053/j.gastro.2010.01.053

[phy213182-bib-0005] Chey, W. D. , J. Kurlander , and S. Eswaran . 2015 Irritable bowel syndrome: a clinical review. JAMA 313:949–958. 25734736 10.1001/jama.2015.0954

[phy213182-bib-0006] Craven, M. , C. E. Egan , S. E. Dowd , S. P. McDonough , B. Dogan , E. Y. Denkers , et al. 2012 Inflammation drives dysbiosis and bacterial invasion in murine models of ileal Crohn's disease. PLoS ONE 7:e41594. 22848538 10.1371/journal.pone.0041594 PMC3404971

[phy213182-bib-0007] Higgins, P. D. R. , and J. F. Johanson . 2004 Epidemiology of constipation in North America: a systematic review. Am. J. Gastroenterol. 99:750–759. 15089911 10.1111/j.1572-0241.2004.04114.x

[phy213182-bib-0008] Honda, K. , and D. R. Littman . 2012 The microbiome in infectious disease and inflammation. Annu. Rev. Immunol. 30:759–795. 22224764 10.1146/annurev-immunol-020711-074937 PMC4426968

[phy213182-bib-0009] Hurst, N. R. , D. M. Kendig , K. S. Murthy , and J. R. Grider . 2014 The short chain fatty acids, butyrate and propionate, have differential effects on the motility of the guinea pig colon. Neurogastroenterol. Motil. 26:1586–1596. 25223619 10.1111/nmo.12425 PMC4438679

[phy213182-bib-0010] Husebye, E. , P. M. Hellström , F. Sundler , J. Chen , and T. Midtvedt . 2001 Influence of microbial species on small intestinal myoelectric activity and transit in germ‐free rats. Am. J. Physiol. Gastrointest. Liver Physiol. 280:G368–G380. 11171619 10.1152/ajpgi.2001.280.3.G368

[phy213182-bib-0011] Jeffery, I. B. , P. W. O'Toole , L. Öhman , M. J. Claesson , J. Deane , E. M. M. Quigley , et al. 2012 An irritable bowel syndrome subtype defined by species‐specific alterations in faecal microbiota. Gut 61:997–1006. 22180058 10.1136/gutjnl-2011-301501

[phy213182-bib-0012] Kamath, P. S. , M. T. Hoepfner , and S. F. Phillips . 1987 Short‐chain fatty acids stimulate motility of the canine ileum. Am. J. Physiol. 253:G427–G433. 3661705 10.1152/ajpgi.1987.253.4.G427

[phy213182-bib-0013] Kashyap, P. C. , A. Marcobal , L. K. Ursell , M. Larauche , H. Duboc , K. A. Earle , et al. 2013 Complex interactions among diet, gastrointestinal transit, and gut microbiota in humanized mice. Gastroenterology 144:967–977. 23380084 10.1053/j.gastro.2013.01.047 PMC3890323

[phy213182-bib-0015] Lyra, A. , T. Rinttilä , J. Nikkilä , L. Krogius‐Kurikka , K. Kajander , E. Malinen , et al. 2009 Diarrhoea‐predominant irritable bowel syndrome distinguishable by 16S rRNA gene phylotype quantification. World J. Gastroenterol. 15:5936–5945. 20014457 10.3748/wjg.15.5936 PMC2795180

[phy213182-bib-0016] Machiels, K. , M. Joossens , J. Sabino , V. De Preter , I. Arijs , V. Eeckhaut , et al. 2014 A decrease of the butyrate‐producing species Roseburia hominis and Faecalibacterium prausnitzii defines dysbiosis in patients with ulcerative colitis. Gut 63:1275–1283. 24021287 10.1136/gutjnl-2013-304833

[phy213182-bib-0017] Malinen, E. , T. Rinttilä , K. Kajander , J. Mättö , A. Kassinen , L. Krogius , et al. 2005 Analysis of the fecal microbiota of irritable bowel syndrome patients and healthy controls with real‐time PCR. Am. J. Gastroenterol. 100:373–382. 15667495 10.1111/j.1572-0241.2005.40312.x

[phy213182-bib-0018] Maukonen, J. , R. Satokari , J. Mättö , H. Söderlund , T. Mattila‐Sandholm , and M. Saarela . 2006 Prevalence and temporal stability of selected clostridial groups in irritable bowel syndrome in relation to predominant faecal bacteria. J. Med. Microbiol. 55:625–633. 16585652 10.1099/jmm.0.46134-0

[phy213182-bib-0019] Muller, P. A. , B. Koscsó , G. M. Rajani , K. Stevanovic , M.‐L. Berres , D. Hashimoto , et al. 2014 Crosstalk between muscularis macrophages and enteric neurons regulates gastrointestinal motility. Cell 158:300–313. 25036630 10.1016/j.cell.2014.04.050 PMC4149228

[phy213182-bib-0020] Nagakura, Y. , Y. Naitoh , T. Kamato , M. Yamano , and K. Miyata . 1996 Compounds possessing 5‐HT3 receptor antagonistic activity inhibit intestinal propulsion in mice. Eur. J. Pharmacol. 311:67–72. 8884238 10.1016/0014-2999(96)00403-7

[phy213182-bib-0022] Parkes, G. C. , N. B. Rayment , B. N. Hudspith , L. Petrovska , M. C. Lomer , J. Brostoff , et al. 2012 Distinct microbial populations exist in the mucosa‐associated microbiota of sub‐groups of irritable bowel syndrome. Neurogastroenterol. Motil. 24:31–39. 22070725 10.1111/j.1365-2982.2011.01803.x

[phy213182-bib-0023] Pimentel, M. , E. E. Soffer , E. J. Chow , Y. Kong , and H. C. Lin . 2002 Lower frequency of MMC is found in IBS subjects with abnormal lactulose breath test, suggesting bacterial overgrowth. Dig. Dis. Sci. 47:2639–2643. 12498278 10.1023/a:1021039032413

[phy213182-bib-0024] Quigley, E. M. M. 2011 The enteric microbiota in the pathogenesis and management of constipation. Best Pract. Res. Clin. Gastroenterol. 25:119–126. 21382583 10.1016/j.bpg.2011.01.003

[phy213182-bib-0025] Renom, G. , P. Bulois , S. Hafraoui , J. F. Colombel , and P. M. Degand . 2001 Simple gas chromatography analysis of faecal butyrate: application to patients at risk of pouchitis. Clin. Chem. Lab. Med. 39:15–19. 11256793 10.1515/CCLM.2001.005

[phy213182-bib-0026] Ridaura, V. K. , J. J. Faith , F. E. Rey , J. Cheng , A. E. Duncan , A. L. Kau , et al. 2013 Gut microbiota from twins discordant for obesity modulate metabolism in mice. Science 341:1241214. 24009397 10.1126/science.1241214 PMC3829625

[phy213182-bib-0027] Rodolphe Soret, R. , J. Chevalier , P. De Coppet , G. Poupeau , P. Derkinderen , J. P. Segain , et al. 2010 Short‐chain fatty acids regulate the enteric neurons and control gastrointestinal motility in rats. Gastroenterology 138:1772–1782. 20152836 10.1053/j.gastro.2010.01.053

[phy213182-bib-0028] Suply, E. , P. de Vries , R. Soret , F. Cossais , and M. Neunlist . 2012 Butyrate enemas enhance both cholinergic and nitrergic phenotype of myenteric neurons and neuromuscular transmission in newborn rat colon. Am. J. Physiol. Gastrointest. Liver Physiol. 302:G1373–G1380. 22492692 10.1152/ajpgi.00338.2011

[phy213182-bib-0029] Vega, A. B. , A. Perelló , L. Martos , I. García Bayo , M. García , V. Andreu , et al. 2015 Breath methane in functional constipation: response to treatment with Ispaghula husk Neurogastroenterol. Motil. 27: 945–953. 25952409 10.1111/nmo.12568

